# O Escore HALP como um Novo Marcador Associado à Circulação Colateral Coronariana em Pacientes com Oclusão Total Crônica

**DOI:** 10.36660/abc.20260206

**Published:** 2026-08-03

**Authors:** Hasan Akkaya, Muhammet Öztürk

**Affiliations:** 1 Department of Cardiology Niğde Training and Research Hospital Niğde Turquia Department of Cardiology, Niğde Training and Research Hospital, Niğde – Turquia

**Keywords:** Circulação Colateral, Oclusão Coronariana, Prognóstico, Inflamação

## Abstract

**Fundamento:**

A circulação colateral coronariana (CCC) é um determinante fundamental da viabilidade miocárdica e do prognóstico em pacientes com oclusão total crônica (OTC). O escore de hemoglobina, albumina, linfócitos e plaquetas (HALP) reflete o estado nutricional, a competência imunológica e a inflamação sistêmica.

**Objetivos:**

Investigar a associação entre o escore HALP e a CCC em pacientes com OTC.

**Métodos:**

Este estudo retrospectivo, unicêntrico, incluiu 269 pacientes com síndrome coronariana crônica e OTC confirmada por angiografia. Os pacientes foram classificados de acordo com os graus de Rentrop e dicotomizados em CCC ruim (Rentrop 0–1; n=119) e CCC boa (Rentrop 2–3; n=150). O escore HALP, marcadores inflamatórios, índices aterogênicos e escores angiográficos foram comparados entre os grupos. Foram realizadas análises de regressão logística multivariada e de curva ROC (característica de operação do receptor). Um valor de p <0,05 foi considerado estatisticamente significativo.

**Resultados:**

O escore HALP aumentou significativamente ao longo dos graus de Rentrop (p<0,001) e foi maior em pacientes com boa consistência interna do que naqueles com consistência interna ruim (75,52±13,91 vs. 35,10±11,29; p<0,001). Na análise multivariada, o escore HALP (OR: 0,88; IC 95%: 0,83–0,93; p<0,001), o escore de Gensini (OR: 1,14; p=0,002) e o Índice Prognóstico Nutricional (OR: 0,73; p=0,003) foram preditores independentes de boa consistência interna. A análise ROC mostrou alta capacidade discriminativa (AUC=0,958; IC 95%: 0,93–0,98), com um valor de corte >49,08 (91,3% de sensibilidade, 90,8% de especificidade). Os índices lipídicos aterogênicos não apresentaram associação significativa com a CCC.

**Conclusões:**

O escore HALP é um marcador independente e facilmente disponível associado à CCC em pacientes com OTC e pode servir como uma ferramenta prática para estratificação de risco, destacando a importância das vias inflamatórias, nutricionais e imunológicas no desenvolvimento de circulação colateral.

## Graphical abstract

**Figura Central:**
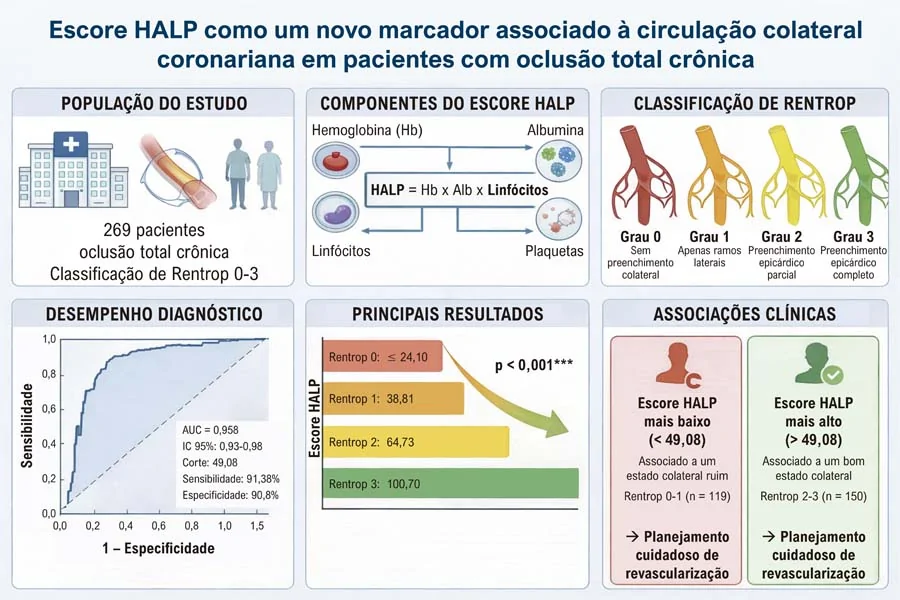
O Escore HALP como um Novo Marcador Associado à Circulação Colateral Coronariana em Pacientes com Oclusão Total Crônica

Um breve resumo visual do estudo.

## Introdução

A doença arterial coronariana continua sendo a principal causa global de mortalidade cardiovascular, sendo a oclusão total crônica (OTC) um subgrupo particularmente desafiador, ocorrendo em 15–30% dos pacientes submetidos à angiografia diagnóstica.^1,2^ A revascularização da OTC permanece tecnicamente complexa, e muitos pacientes recebem apenas terapia medicamentosa otimizada; portanto, a adequação da circulação colateral coronariana (CCC) torna-se um determinante crítico da viabilidade miocárdica e do prognóstico a longo prazo.^3,4^

A CCC engloba condutos vasculares pré-existentes ou recém-formados que contornam segmentos ocluídos, com marcada variabilidade interindividual, que varia de praticamente ausente (Rentrop 0) a fluxo compensatório completo (Rentrop 3).^5^ Colaterais bem desenvolvidas conferem infartos de menor tamanho, melhor preservação da função ventricular esquerda e maior sobrevida,^6-8^enquanto a CCC inadequada está associada a áreas isquêmicas maiores e aumento da mortalidade.^9^

A formação de colaterais coronarianas ocorre principalmente por meio da arteriogênese — remodelação e alargamento de conexões arteriolares preexistentes, impulsionados pelo estresse de cisalhamento hemodinâmico resultante dos gradientes de pressão através de segmentos estenóticos.^10^ No entanto, a arteriogênese é profundamente influenciada pelo estado inflamatório sistêmico, função imunológica, estado nutricional e parâmetros hematológicos.^1 1^ A inflamação crônica de baixo grau prejudica a função endotelial e inibe a formação de colaterais,^1 2^ enquanto respostas imunes equilibradas promovem o reparo endotelial.^1 3^ Além disso, a desnutrição e a hipoalbuminemia comprometem a integridade vascular, enquanto a anemia limita o fornecimento de oxigênio, atenuando as vias do fator induzível por hipóxia.^14,15^

Índices inflamatórios e marcadores nutricionais têm sido associados a desfechos coronarianos,^16-20^ e estudos anteriores relacionaram a Razão Neutrófilo-Linfócito (RNL) e a Razão Plaqueta-Linfócito (RPL) à CCC.^2 1,2 2^ No entanto, esses índices de parâmetro único capturam apenas aspectos parciais da interação inflamatória-nutricional que governa a arteriogênese.

O escore hemoglobina, albumina, linfócitos e plaquetas (HALP) — calculado como (Hemoglobina × Albumina × contagem de Linfócitos) / contagem de Plaquetas — integra quatro componentes-chave que refletem a capacidade de transporte de oxigênio, o estado nutricional, a competência imunológica e a atividade hemostática-inflamatória.^23,24^ Recentemente, o escore HALP demonstrou relevância cardiovascular, com escores mais baixos associados independentemente a maior mortalidade por infarto agudo do miocárdio^25^ e eventos adversos pós-intervenção.^26^ No entanto, nenhum estudo anterior investigou a relação do escore HALP com a CCC.

A avaliação tradicional do risco cardiovascular com base em lipídios depende de parâmetros como colesterol LDL, colesterol HDL e triglicerídeos. Evidências recentes sugerem que índices aterogênicos compostos — incluindo o Índice Aterogênico do Plasma, os Índices de Risco de Castelli, o Coeficiente Aterogênico e o Índice de Triglicerídeos-Glicose — podem refletir melhor a desregulação metabólica.^27,28^ No entanto, sua associação com o desenvolvimento de colaterais coronarianas permanece pouco caracterizada, deixando em aberto se a aterogênese induzida por lipídios influencia diretamente a capacidade arteriogênica ou se a colateralização é governada principalmente por fatores hemodinâmicos e inflamatórios independentes do metabolismo lipídico.

Considerando o papel crucial da CCC em pacientes com OTC e a escassez de biomarcadores integrativos, elaboramos este estudo para avaliar de forma abrangente as associações entre o escore HALP, parâmetros inflamatórios, índices aterogênicos e CCC. Até onde sabemos, este é o primeiro estudo a investigar a associação do escore HALP com a CCC.

## Métodos

### Desenho do estudo e população

Este estudo retrospectivo, observacional e unicêntrico foi conduzido em Revisão cega. O protocolo do estudo foi aprovado pelo Comitê de Ética Institucional de Revisão cega e seguiu os princípios da Declaração de Helsinque. Devido à natureza retrospectiva e à utilização de dados anonimizados, a exigência de consentimento informado foi dispensada.

Foram selecionados pacientes com idade ≥18 anos diagnosticados com síndrome coronariana crônica (SCC) de acordo com as diretrizes atuais^29^ que realizaram angiografia coronariana entre agosto de 2021 e janeiro de 2026. Os critérios de inclusão compreenderam: angina típica, evidência de isquemia eletrocardiográfica, cintilografia de perfusão miocárdica positiva ou estenose crítica na angiotomografia coronariana; além de oclusão total documentada angiograficamente (estenose de 100%, fluxo TIMI 0) em ≥1 artéria coronariana epicárdica principal.

Os critérios de exclusão incluíram: síndrome coronariana aguda nos 6 meses anteriores; cirurgia prévia de revascularização do miocárdio; doença valvar moderada a grave; TFGe <30 ml/min/1,73m^2^; doença hepática ativa; neoplasia maligna ativa conhecida; insuficiência cardíaca classe III-IV da NYHA; doença pulmonar obstrutiva crônica moderada a grave; doença infecciosa ou reumatológica aguda/crônica; ou dados incompletos.

### Coleta de dados e avaliação laboratorial

Os dados demográficos, clínicos, laboratoriais e angiográficos basais foram extraídos retrospectivamente dos registros eletrônicos. Os fatores de risco cardiovascular e os medicamentos foram documentados no momento da angiografia.

Amostras de sangue venoso foram coletadas após jejum noturno (≥8 horas) nas manhãs dos exames de angiografia. Os parâmetros laboratoriais incluíram: hemograma completo (hemoglobina, RDW, leucócitos, neutrófilos, linfócitos, monócitos, plaquetas); parâmetros bioquímicos (glicemia de jejum, creatinina, AST, ALT, PCRas, HbA1c, colesterol total, HDL-C, LDL-C, triglicerídeos, albumina). A TFGe foi calculada utilizando a equação CKD-EPI.

### Cálculo de índices compostos

- **Escore HALP** = (Hemoglobina [g/L] × Albumina [g/dL] × Contagem de linfócitos [×10^9^/L]) / Contagem de plaquetas (×10^9^/L).
- **Índice CALLY** = Albumina × Contagem de linfócitos / Proteína C-reativa
- Í**ndice de Resposta Inflamatória Sistêmica (SIRI)** = Contagem de neutrófilos × Contagem de monócitos / Contagem de linfócitos
- **Índice de Imunoinflamação Sistêmica (SII)** = Contagem de plaquetas × Contagem de neutrófilos / Contagem de linfócitos
- **Índice Prognóstico Nutricional (IPN)** = 10 × Albumina + 0,005 × Contagem de linfócitos
- **Relação plaquetas-linfócitos (RPL)** = Contagem de plaquetas / Contagem de linfócitos
- **Relação Neutrófilo-Linfócito (RNL)** = Contagem de neutrófilos / Contagem de linfócitos
- **Relação Monócito-Linfócito (RML)** = Contagem de monócitos / Contagem de linfócitos
- **Valor de inflamação pan-imune (PIV)** = Neutrófilo × Plaqueta × Monócito / Linfócito
- **Índice Aterogênico do Plasma (IAP)** = Log_10_ (Triglicerídeos / Colesterol HDL)
- **Índice de Risco Castelli I (IC-I)** = Colesterol total / Colesterol HDL
- **Índice de Risco Castelli II (IC-II)** = Colesterol LDL / Colesterol HDL
- **Coeficiente Aterogênico (CA)** = (Colesterol total − Colesterol HDL) / Colesterol HDL
- **Índice de Triglicerídeos-Glicose (TyG)** = Ln (Triglicerídeos × Glicemia em jejum / 2)

### Angiografia coronariana e avaliação de colaterais

A angiografia coronariana foi realizada por via femoral ou radial. Dois cardiologistas intervencionistas experientes, sem conhecimento dos dados clínico-laboratoriais, revisaram as imagens angiográficas; as discordâncias foram resolvidas por um terceiro cardiologista sênior. Os escores SYNTAX e Gensini foram calculados utilizando métodos padrão. A CCC foi avaliada pela classificação de Rentrop (0–3).^5^ Os pacientes foram divididos em dois grupos: CCC ruim (Rentrop 0–1) e CCC boa (Rentrop 2–3) para análises secundárias.

### Análise estatística

As análises estatísticas foram realizadas utilizando o IBM SPSS Statistics versão 22.0 e o MedCalc versão 22.018. Um valor de p bicaudal <0,05 foi considerado estatisticamente significativo. A normalidade das variáveis contínuas foi testada utilizando o teste de Shapiro-Wilk. As variáveis com distribuição normal são apresentadas como média ± desvio padrão; as variáveis categóricas, como frequências e percentagens. As variáveis categóricas foram comparadas utilizando o teste χ^2^ ou o teste exato de Fisher. As variáveis contínuas foram comparadas utilizando o teste-t de Student para amostras independentes ou ANOVA de uma via com correção de Bonferroni. O coeficiente de correlação de Pearson avaliou as associações lineares entre as variáveis contínuas. A regressão logística binária univariada identificou as variáveis associadas a uma boa CCC. As variáveis com p<0,10 na análise univariada, juntamente com covariáveis clinicamente relevantes (sexo, fibrilação atrial, fração de ejeção do ventrículo esquerdo (FEVE)), foram incluídas na regressão logística binária multivariada utilizando o método de entrada. Sexo, fibrilação atrial e FEVE foram incluídos como covariáveis devido às suas associações significativas com o estadiamento de Rentrop na análise basal (p=0,004, p=0,001 e p=0,005, respectivamente), refletindo relações biológicas conhecidas entre essas variáveis e a capacidade arteriogênica. A multicolinearidade foi avaliada utilizando o fator de inflação da variância (FIV) (a); FIV >5 indicou multicolinearidade significativa. Os valores de FIV para o modelo final de três variáveis foram: escore HALP 7,13, escore de Gensini 7,90 e IPN 19,38. Embora o IPN tenha apresentado um FIV >5, ele foi mantido devido à sua relevância biológica estabelecida e significância independente; análises de sensibilidade com um modelo de duas variáveis (HALP + Gensini) confirmaram a robustez dos achados. A qualidade do ajuste do modelo foi avaliada utilizando o teste de Hosmer-Lemeshow (p>0,05 indicando bom ajuste). Os resultados são apresentados como razões de chances com intervalos de confiança de 95%. A análise da curva ROC avaliou a capacidade discriminatória do escore HALP para predizer uma boa CCC. A validação interna foi realizada utilizando reamostragem *bootstrap* (1.000 iterações). A AUC corrigida para otimismo foi de 0,967 (IC 95% 0,958–0,969), confirmando a estabilidade do modelo e a ausência de sobreajuste significativo. O ponto de corte ideal foi determinado utilizando o índice de Youden.

## Resultados

### Características basais

Um total de 347 pacientes com OTC submetidos a angiografia coronariana para SCC foram triados. Setenta e oito pacientes foram excluídos devido a síndrome coronariana aguda nos últimos 6 meses (n=18), cirurgia prévia de revascularização miocárdica (CRM) (n=12), taxa de filtração glomerular estimada (TFGe) <30 mL/min/1,73 m^2^ (n=15), neoplasia maligna ativa (n=8) ou dados incompletos (n=25). Os 269 pacientes restantes com SCC e ≥1 artéria coronariana totalmente ocluída foram incluídos (ver Figura 1 para o fluxograma dos pacientes). Os pacientes foram estratificados pela classificação de Rentrop: Rentrop 0 (n=30, 11,2%), Rentrop 1 (n=89, 33,1%), Rentrop 2 (n=105, 39,0%) e Rentrop 3 (n=45, 16,7%). Para análises posteriores, os pacientes foram divididos em dois grupos: aqueles com CCC ruim (Rentrop 0–1; n=119, 44,2%) e aqueles com CCC boa (Rentrop 2–3; n=150, 55,8%).

**Figura 1:**
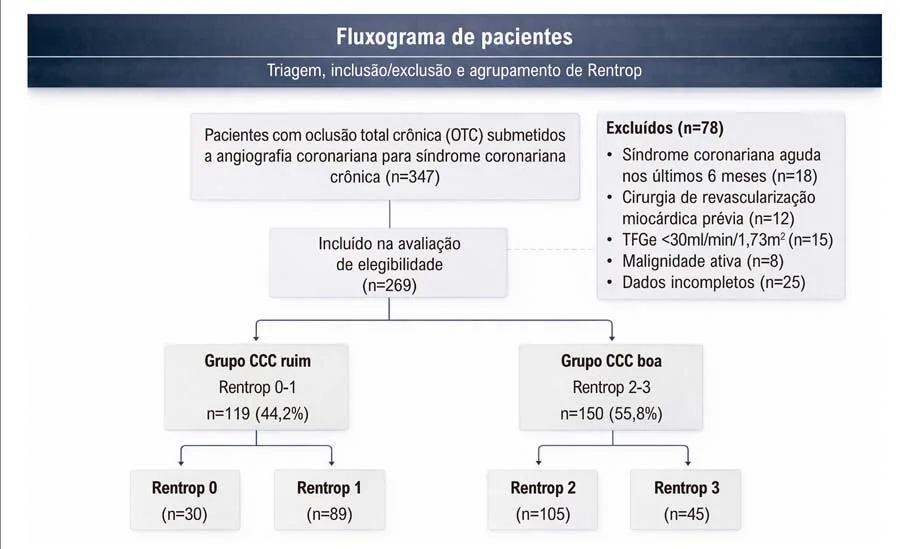
– Diagrama de fluxo de pacientes. CCC: circulação colateral coronariana.

As características demográficas e clínicas basais em relação aos estágios de Rentrop são apresentadas na Tabela 1. Foram observadas associações significativas entre os estágios de Rentrop e o sexo (p=0,004) e a prevalência de fibrilação atrial (p=0,001). Comparações detalhadas entre os grupos são apresentadas na Tabela 1. Não foram observadas diferenças significativas em relação a diabetes mellitus, hipertensão, dislipidemia, tabagismo atual ou uso de medicamentos (p>0,05 para todos).

**Tabela 1:** – Características demográficas e clínicas basais de acordo com os estágios de Rentrop

Característica	Rentrop 0 (n=30)	Rentrop 1 (n=89)	Rentrop 2 (n=105)	Rentrop 3 (n=45)	p
**Idade (anos)**	72,83±9,50*	68,17±11,54	64,75±10,29	62,44±10,26	<0,001
**Sexo masculino, n (%)**	19 (63,3)	59 (66,3)	86 (81,9)	40 (88,9)	0,004
**IMC (kg/m^2^)**	28,22±4,63	27,64±3,59	26,91±3,53	27,61±3,35	0,272
**Fatores de risco, n (%)**					
Diabetes mellitus	12 (40,0)	34 (38,2)	36 (34,3)	16 (35,6)	0,917
Hipertensão	20 (66,7)	52 (58,4)	61 (58,1)	24 (53,3)	0,724
Dislipidemia	14 (46,7)	36 (40,4)	52 (49,5)	18 (40,0)	0,554
Fumante atual	12 (40,0)	38 (42,7)	41 (39,0)	27 (60,0)	0,113
Fibrilação atrial	5 (16,7)	9 (10.1)	4 (3,8)	0 (0,0)	0,001
**Medicamentos, n(%)**					
Betabloqueador	20 (66,7)	53 (59,6)	57 (54,3)	26 (57,8)	0,658
IECA/BRA	14 (46,7)	35 (39,3)	47 (44,8)	20 (44,4)	0,844
Estatina	12 (40,0)	24 (27,0)	34 (32,4)	14 (31.1)	0,595
Bloqueador dos canais de cálcio	9 (30,0)	18 (20.2)	19 (18.1)	8 (17,8)	0,523

Os dados são apresentados como média ± desvio padrão ou n (%). Valores de p: ANOVA de uma via ou teste χ^2^. *p<0,05 vs Rentrop 2 e Rentrop 3 (correção de Bonferroni). IMC: índice de massa corporal; IECA: inibidor da enzima conversora de angiotensina; BRA: bloqueador do receptor de angiotensina.

### Parâmetros laboratoriais e angiográficos

A análise comparativa entre os estágios de Rentrop revelou diferenças significativas em múltiplos parâmetros clínicos, hematológicos, inflamatórios e angiográficos (Tabela 2). As principais diferenças clínicas estão resumidas na Tabela 2. Notavelmente, a idade, a FEVE e a TFGe apresentaram diferenças significativas entre os estágios de Rentrop (p<0,001, p=0,005 e p=0,022, respectivamente), enquanto os parâmetros bioquímicos não apresentaram diferenças significativas (p>0,05 para todos).

**Tabela 2:** – Parâmetros laboratoriais, hematológicos, inflamatórios e escores angiográficos de acordo com os estágios de Rentrop

Parâmetro	Rentrop 0 (n=30)	Rentrop 1 (n=89)	Rentrop 2 (n=105)	Rentrop 3 (n=45)	p
**Clínico/Bioquímico**					
FEVE (%)	45,80±10,15*	49,19±8,27	50,58±6,48	51,82±6,38	0,005
TFGe (ml/min/1,73m^2^)	71,33±16,99†	77,55±14,63	82,69±13,67	87,12±14,95	0,022
Creatinina (mg/dL)	1,21±0,91	1,05±0,50	1,03±0,51	0,97±0,30	0,338
AST (U/L)	27,57±11,27	24,27±9,67	23,77±9,28	23,00±7,58	0,391
ALT (U/L)	19,37±9,54	19,94±8,65	19,95±9,14	18,78±8,81	0,917
Albumina (g/dL)	38,82±3,46	40,51±3,42	42,57±2,76	43,36±2,82‡	<0,001
PCRas (mg/L)	5,95±4,59	5,46±1,49	3,97±4,58	4,76±7,56	0,131
**Hematológico**					
Hemoglobina (g/L)	12,35±2,36	13,68±1,89	14,56±1,84	14,92±1,36‡	<0,001
WBC (×10^9^/L)	9,24±2,85	9,08±2,98	9,02±2,52	9,51±2,32	0,770
Neutrófilo (×10^9^/L)	6,84±2,49	6,38±2,60	5,72±2,23	5,05±1,37*	0,002
Linfócitos (×10^9^/L)	1,61±0,59	1,94±0,73	2,43±0,72	3,49±1,29‡	<0,001
Monócito (×10⁹/L)	0,61±0,25	0,60±0,24	0,66±0,23	0,73±0,22	0,015
Plaquetas (×10^9^/L)	300,40±58,65*	272,06±47,63	243,47±51,33	225,47±53,74	<0,001
**Índices inflamatórios/nutricionais**					
Escore HALP	24,10±11,30	38,81±10,44	64,73±15,91	100,70±16,37‡	<0,001
Índice CALLY	26,07±12,87	26,70±11,55	53,54±13,45	99,31±18,31‡	<0,001
SII	1837,27±329,81	1042,91±261,02	646,64±126,33	361,26±59,66	<0,001
IPN	37,73±7,32	40,04±6,51	42,65±2,76	43,29±2,85	<0,001
RPL	246,02±15,62	149,49±14,98	109,61±12,11	72,00±10,64‡	<0,001
RNL	6,03±2,71‡	3,66±1,55	2,82±0,35	1,72±0,87	<0,001
RML	0,48±0,21‡	0,34±0,14	0,32±0,12	0,23±0,09	0,001
SIRI	3,65±0,37	2,54±0,24	2,18±1,41	1,19±0,63	0,077
PIV	1089,06±157,96	773,98±106,71	512,71±89,36	265,74±62,67	0,047
**Índices aterogênicos**					
IAP	0,15±0,11	0,21±0,18	0,26±0,14	0,24±0,11	0,577
IC-I	4,46±1,20	4,69±1,03	4,83±1,17	4,98±1,41	0,223
IC-II	2,81±0,47	2,98±0,94	2,97±0,96	3,04±1,20	0,805
Índice TyG	9,18±0,71	9,24±0,59	9,30±0,58	9,21±0,53	0,700
**Escores angiográficos**					
Escore SINTAX	26,58±7,24‡	22,71±6,59	18,12±5,61	16,84±6,78	<0,001
Escore Gensini	59,60±18,87	53,09±16,07*	36,87±12,11	31,49±13,77	<0,001

Os dados são apresentados como média ± desvio padrão. Valores de p: ANOVA de uma via com correção post-hoc de Bonferroni. *p<0,05 vs Rentrop 3; †p<0,05 vs Rentrop 3; ‡p<0,05 vs todos os outros grupos. FEVE: fração de ejeção do ventrículo esquerdo; TFGe: taxa de filtração glomerular estimada; AST: aspartato aminotransferase; ALT: alanina aminotransferase; PCR-as: proteína C-reativa de alta sensibilidade; WBC: glóbulos brancos; SII: índice sistêmico de imuno-inflamação; IPN: índice prognóstico nutricional; RPL: relação plaqueta-linfócito; RNL: relação neutrófilo-linfócito; RML: relação monócito-linfócito; SIRI: índice de resposta inflamatória sistêmica; PIV: valor de inflamação pan-imune; IAP: índice aterogênico do plasma; IC: índice de risco de Castelli; TyG: índice triglicerídeo-glicose.

Os parâmetros hematológicos mostraram diferenças marcantes e graduais entre os estágios de Rentrop (Tabela 2). Os níveis de albumina, hemoglobina e a contagem de linfócitos aumentaram progressivamente com a melhora da circulação colateral, enquanto as contagens de neutrófilos e plaquetas diminuíram (todos com p<0,001).

O escore HALP demonstrou um gradiente gradual notável ao longo dos estágios de Rentrop (p<0,001), com o Rentrop 3 apresentando valores significativamente mais altos em comparação com todos os outros grupos. O índice CALLY e o IPN mostraram aumentos graduais semelhantes (Tabela 2).

Os índices inflamatórios mostraram relações inversas com a circulação colateral. SII, RPL, RNL, RML e PIV foram todos significativamente maiores em pacientes com CCC ruim (Tabela 2). O SIRI apresentou uma tendência à redução com melhor circulação colateral (p=0,077).

A complexidade angiográfica foi maior em pacientes com CCC ruim, com escores SYNTAX e Gensini mais elevados (Tabela 2; p<0,001 para ambos).

Os índices lipídicos aterogênicos, incluindo IAP, IC-I, IC-II, CA e índice TyG, não apresentaram diferenças significativas entre os estágios de Rentrop (p>0,05 para todos), sugerindo que os fatores de risco cardiovascular tradicionais baseados em lipídios não influenciam independentemente o desenvolvimento da CCCs.

### Comparação entre CCC ruim e bom

Em consonância com a literatura anterior, a CCC foi categorizada em CCC ruim (Rentrop 0–1; n=119) e CCC boa (Rentrop 2–3; n=150). Comparações detalhadas são apresentadas na Tabela 3. Pacientes com CCC ruim apresentaram escores significativamente mais elevados de HALP, CALLY e IPN, enquanto marcadores inflamatórios (SII, RPL, RNL, RML, PIV e razão PCRas/Albumina) estavam significativamente elevados em pacientes com CCC ruim (todos p<0,05).

**Tabela 3:** – Comparação dos parâmetros entre os grupos com circulação colateral coronariana ruim e boa

Parâmetro	CCC ruim (n=119)	CCC Boa (n=150)	p
**Escores angiográficos**			
Escore SINTAX	23,68±6,73	17,74±6,08	<0,001
Escore Gensini	54,73±16,97	35,25±13,14	<0,001
**Índices inflamatórios e nutricionais**			
Escore HALP	35,10±11,29	75,52±13,91	<0,001
Índice CALLY	26,67±4,57	68,30±12,63	<0,001
SIRI	2,82±1,44	1,88±0,39	0,065
SII	1243,17±35,88	561,03±25,99	<0,001
IPN	39,46±5,81	42,84±2,80	<0,001
RPL	173,82±10,04	98,33±8,57	<0,001
RNL	4,26±1,83	2,49±0,66	<0,001
RML	0,37±0,18	0,29±0,11	0,014
Relação PCRas/Albumina	0,14±0,06	0,10±0,02	0,007
PIV	853,41±99,14	438,62±63,91	0,016
**Índices aterogênicos**			
IAP	2,84±0,25	0,26±0,05	0,331
IC-I	4,63±1,07	4,88±1,25	0,083
IC-II	2,94±0,94	2,99±1,04	0,657
CA	3,61±1,06	3,86±1,25	0,076
Índice TyG	9,22±0,62	9,27±0,57	0,492

Os dados são apresentados como média ± desvio padrão. Valores de p: teste t de amostras independentes. CCC: circulação colateral coronariana; SIRI: índice de resposta inflamatória sistêmica; SII: índice de imunoinflamação sistêmica; IPN: índice prognóstico nutricional; RPL: relação plaqueta-linfócito; RNL: relação neutrófilo-linfócito; RML: relação monócito-linfócito; PCRas: proteína C-reativa de alta sensibilidade; PIV: valor de inflamação pan-imune; IAP: índice aterogênico do plasma; IC: índice de risco de Castelli; CA: coeficiente aterogênico; TyG: índice triglicerídeo-glicose.

A complexidade angiográfica foi maior em pacientes com CCC ruim (Tabela 3). Os índices aterogênicos não diferiram significativamente entre os grupos (p>0,05 para todos).

### Análise de correlação

A análise de correlação de Pearson (Tabela 4) revelou associações significativas entre o escore HALP e múltiplos parâmetros. O escore HALP demonstrou fortes correlações negativas com o RPL (r = −0,670, p < 0,001), correlações negativas moderadas com o escore de Gensini (r = −0,517), SII (r = −0,451), escore SYNTAX (r = −0,439) e RNL (r = −0,418), e correlações negativas fracas com RML, PIV e SIRI (todos com p < 0,001). Correlações positivas foram observadas com o índice CALLY (r = +0,413) e o IPN (r = +0,292). Os valores detalhados são apresentados na Tabela 4.

**Tabela 4:** – Correlação entre o Escore HALP e os Parâmetros do Estudo

Parâmetro	r	p
**Escores angiográficos**		
Escore SINTAX	–0,439	<0,001
Escore Gensini	–0,517	<0,001
**Índices inflamatórios e nutricionais**		
Índice CALLY	+0,413	<0,001
SIRI	–0,209	0,001
SII	–0,451	<0,001
IPN	+0,292	<0,001
RPL	–0,670	<0,001
RNL	–0,418	<0,001
RML	–0,294	<0,001
PIV	–0,232	<0,001
Relação PCRas/Albumina	–0,105	0,087
**Índices aterogênicos**		
IAP	–0,075	0,218
IC-I	+0,123	0,043
IC-II	+0,088	0,151
CA	+0,125	0,040
Índice TyG	–0,020	0,745

Análise de correlação de Pearson. SIRI: índice de resposta inflamatória sistêmica; SII: índice de imunoinflamação sistêmica; IPN: índice prognóstico nutricional; RPL: relação plaquetas/linfócitos; RNL: relação neutrófilos/linfócitos; RML: relação monócitos/linfócitos; PIV: valor de inflamação pan-imune; PCRas: proteína C-reativa de alta sensibilidade; IAP: índice aterogênico do plasma; CRI: índice de risco de Castelli; CA: coeficiente aterogênico; TyG: índice triglicerídeo-glicose.

### Análise de regressão logística

A regressão logística univariada identificou múltiplos parâmetros significativamente associados ao bom desenvolvimento da CCC (Tabela 5). Após o ajuste para potenciais fatores de confusão no modelo multivariável, apenas três parâmetros permaneceram como associados independentes ao bom desenvolvimento da CCC: escore HALP (OR 0,88, IC 95% 0,83–0,93, p<0,001), escore de Gensini (OR 1,14, IC 95% 1,05–1,24, p=0,002) e IPN (OR 0,73, IC 95% 0,60–0,90, p=0,003).

**Tabela 5:** – Análise de regressão logística univariada e multivariada para preditores de boa circulação colateral coronariana

Variável	OR univariada (IC 95%)	p	OR multivariada (IC 95%)	p
Escore HALP	0,86 (0,83–0,89)	<0,001	0,88 (0,83–0,93)	<0,001
Escore Gensini	1,08 (1,06–1,10)	<0,001	1,13 (1,05–1,22)	0,002
IPN	0,76 (0,69–0,83)	<0,001	0,73 (0,60–0,90)	0,003
Idade (anos)	1,04 (1,02–1,07)	<0,001	1,02 (0,97–1,07)	0,388
Escore SINTAX	1,15 (1,10–1,20)	<0,001	0,85 (0,69–1,04)	0,128
Índice CALLY	0,98 (0,97–0,99)	<0,001	0,99 (0,98–1,01)	0,515
SII	1,00 (0,99–1,03)	<0,001	1,00 (0,99–1,01)	0,084
RPL	1,03 (1,02–1,04)	<0,001	0,99 (0,97–1,02)	0,676
RNL	1,42 (1,22–1,66)	<0,001	0,57 (0,21–1,53)	0,261
Relação PCRas/Albumina	19,67 (2,05–188,98)	0,009	27,06 (0,17–44,61)	0,201
SIRI	1,13 (0,97–1,31)	0,102	—	—
IAP	1,01 (0,96–1,07)	0,602	—	—

Variável dependente: Circulação colateral coronariana deficiente (Rentrop 0-1=1, boa CCC=0). Os valores em negrito indicam preditores independentes na análise multivariável. OR<1 para o escore HALP indica que escores HALP mais altos estão associados a menores chances de CCC deficiente (efeito protetor). Valores de FIV para o modelo final: HALP=7,13, Gensini=7,90, IPN=19,38. Validação interna por bootstrap (1.000 iterações): AUC corrigida para otimismo=0,958 (IC 95%: 0,929-0,980). OR: odds ratio; IC: intervalo de confiança; IPN: índice prognóstico nutricional; SII: índice sistêmico de imunoinflamação; RPL: relação plaqueta-linfócito; RNL: relação neutrófilo-linfócito; SIRI: índice de resposta inflamatória sistêmica; PCRas: proteína C-reativa de alta sensibilidade; IAP: índice aterogênico do plasma.

A variável dependente foi codificada como CCC boa = 1 (Rentrop 2–3) e CCC ruim = 0 (Rentrop 0–1). A razão de chances (OR) de 0,88 para o escore HALP indica que, para cada aumento de uma unidade no escore HALP, a probabilidade de ser classificado como tendo boa CCC aumenta (ou seja, a probabilidade de CCC ruim diminui em 12%). Isso é consistente com a hipótese biológica: escores HALP mais altos estão associados a melhor colateralização. A aparente OR inversa reflete a escala da variável HALP em relação à codificação binária do resultado.

Uma regressão logística ordinal adicional, utilizando o desfecho completo de Rentrop em 4 graus, confirmou a associação independente do escore HALP (OR 0,91, IC 95% 0,87–0,95, p<0,001), do Escore Gensini (OR 1,09, IC 95% 1,02–1,17, p=0,012) e do IPN (OR 0,81, IC 95% 0,68–0,96, p=0,016) com o grau de Rentrop, corroborando a robustez dos achados em todo o espectro ordinal.

### Análise ROC

A análise da curva ROC demonstrou que o escore HALP possuía excelente poder discriminatório para predizer uma boa CCC (Figura 2). A AUC foi de 0,958 (IC 95% 0,93–0,98, p<0,001). A validação interna utilizando reamostragem *bootstrap* (1.000 iterações) resultou em uma AUC corrigida para otimismo de 0,967 (IC 95% 0,958–0,969), confirmando a estabilidade do modelo e a ausência de sobreajuste significativo. O ponto de corte ideal >49,08 apresentou sensibilidade de 91,3% (IC 95% 85,6–95,3%) e especificidade de 90,8% (IC 95% 84,1–95,3%).

**Figura 2:**
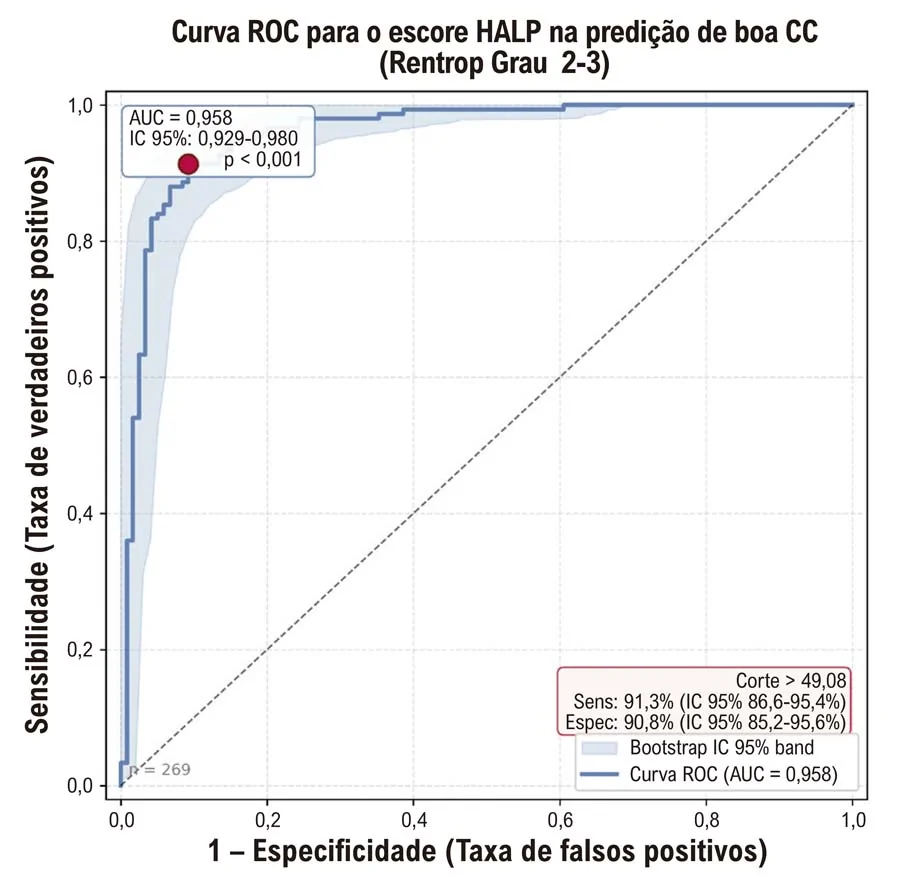
– Curva ROC (Receiver Operating Characteristic) para o escore HALP na predição de boa circulação colateral coronariana.

## Discussão

Este estudo investigou as relações entre o escore HALP, parâmetros inflamatórios, índices aterogênicos e CCC em pacientes com OTC. Nossos principais achados demonstram: (1) o escore HALP apresentou um aumento progressivo robusto ao longo dos estágios de Rentrop; (2) os índices inflamatórios estavam significativamente elevados em pacientes com CCC ruim; (3) os índices lipídicos aterogênicos não diferiram entre os estágios de Rentrop; (4) na análise multivariada, o escore HALP, o escore de Gensini e o IPN emergiram como associados independentes à boa CCC; e (5) o ponto de corte do escore HALP >49,08 demonstrou excelente capacidade discriminatória (AUC = 0,958), confirmada pela validação interna por *bootstrap* (AUC corrigida para otimismo = 0,967). Um resumo visual é apresentado na Figura Central.

O escore HALP integra hemoglobina (capacidade de transporte de oxigênio), albumina (estado nutricional e propriedades anti-inflamatórias), linfócitos (competência imunológica adaptativa) e plaquetas (atividade hemostática-inflamatória). Níveis mais elevados de hemoglobina favorecem a atividade angiogênica por meio das vias HIF-1α/VEGF,^30^ enquanto níveis adequados de albumina preservam a função endotelial e a biodisponibilidade do óxido nítrico.^15,31^ Os linfócitos orquestram o reparo vascular por meio da mobilização de células progenitoras endoteliais mediada por citocinas,^1 3^ e contagens mais baixas de plaquetas em pacientes com boa circulação colateral podem refletir uma menor ativação inflamatória crônica.^3 2^

Estudos limitados exploraram o escore HALP em contextos cardiovasculares. Xu et al.^25^ relataram que escores HALP mais baixos estavam independentemente associados a uma maior mortalidade por infarto agudo do miocárdio, enquanto Liu et al.^26^ demonstraram que o escore HALP predizia o fenômeno de *no-reflow* e o prognóstico a longo prazo em pacientes com infarto do miocárdio com supradesnivelamento do segmento ST submetidos à intervenção coronária percutânea primária. Nossos achados estendem essas observações ao desenvolvimento de circulação colateral coronária. A *odds ratio* (OR) ajustada multivariada de 0,88 (IC 95%: 0,83–0,93) indica que cada aumento de uma unidade no escore HALP confere uma redução de 12% na probabilidade de CCC insatisfatória.

Diversos índices inflamatórios apresentaram associação inversa com o desenvolvimento de colaterais. SII, RNL, RPL e PIV estavam significativamente elevados em pacientes com CCC ruim, o que está de acordo com evidências de que a inflamação sistêmica exacerbada prejudica a função endotelial e inibe a arteriogênese.^1 2^ Nossos resultados corroboram achados anteriores de Açar et al.^2 1^ e Kurtul et al.,^2 2^ ampliando essas observações ao demonstrar que o escore HALP pode servir como um marcador mais integrativo, capturando tanto a carga inflamatória quanto a reserva nutricional-imune.

O IPN emergiu como um bom associado independente à CCC em nosso modelo multivariável (OR 0,73, IC 95% 0,60–0,90, p=0,003), consistente com dados recentes de Keskin et al.^20^ A ausência de associações significativas entre índices lipídicos aterogênicos e CCC sugere que a arteriogênese é governada principalmente por mecanismos hemodinâmico-inflamatórios, e não por mecanismos baseados em lipídios,^10^ corroborado por estudos em animais que mostram que a hipercolesterolemia não necessariamente prejudica o crescimento colateral.^3 3^

O escore de Gensini emergiu como um associado independente à boa CCC (OR 1,14, IC 95% 1,05–1,24, p=0,002), consistente com o conceito de que a estenose crônica progressiva cria gradientes hemodinâmicos sustentados que estimulam a arteriogênese.^8^ O escore de Gensini, que leva em consideração a gravidade e a extensão da lesão, pode capturar melhor a cronicidade da aterosclerose em comparação com o escore SYNTAX.

A Escore HALP, por ser um biomarcador simples e facilmente calculável, pode representar um adjuvante útil para a estratificação de risco em pacientes com OTC. Pacientes com escores HALP mais baixos podem representar um subgrupo de maior risco que justifica um acompanhamento mais rigoroso; no entanto, dada a natureza observacional e retrospectiva deste estudo, esses achados devem ser considerados como geradores de hipóteses e requerem validação prospectiva antes de embasar a tomada de decisões clínicas.

Nossos resultados sugerem que intervenções destinadas a melhorar os componentes individuais do escore HALP podem promover a formação de colaterais. Suplementação nutricional (para aumentar os níveis de albumina), correção da anemia (para otimizar a hemoglobina), terapias imunomoduladoras (para melhorar a função dos linfócitos) e estratégias antiplaquetárias equilibradas para evitar a supressão excessiva de plaquetas podem, coletivamente, aumentar a capacidade arteriogênica. Evidências preliminares de ensaios randomizados sugerem que a administração do fator estimulador de colônias de granulócitos-macrófagos pode aumentar o desenvolvimento de colaterais por meio da mobilização de monócitos e células progenitoras endoteliais,^34^ embora sejam necessários estudos confirmatórios de maior porte.

Nosso estudo destaca a interação crucial entre inflamação, estado nutricional e função imunológica na regulação da remodelação cardiovascular. Essa mudança de paradigma — de considerar a aterosclerose exclusivamente como uma doença impulsionada por lipídios para reconhecê-la como um distúrbio inflamatório-imune — tem profundas implicações para o desenvolvimento terapêutico. Agentes anti-inflamatórios emergentes, como o canaquinumabe (anti-IL-1β) e o metotrexato em baixa dose, mostraram-se promissores na redução de eventos cardiovasculares,^35^ e nossos achados sugerem que eles podem exercer efeitos benéficos ao preservar a função colateral.

### Limitações

Diversas limitações merecem ser reconhecidas. Primeiro, o desenho retrospectivo e unicêntrico pode limitar a generalização dos resultados e introduzir viés de seleção. Segundo, a avaliação da CCC baseou-se exclusivamente na classificação angiográfica de Rentrop. Terceiro, não medimos o escore HALP em série ao longo do tempo, o que impede a análise da dinâmica temporal e da causalidade. Quarto, a presença de fatores de confusão residuais não pode ser totalmente descartada, apesar do ajuste multivariável. Quinto, a população do estudo foi limitada a pacientes com OTC. Sexto, as unidades de albumina (g/dL) utilizadas em nossa fórmula HALP diferem de algumas fórmulas publicadas que utilizam g/L; os leitores devem aplicar a mesma convenção de unidades ao utilizar o nosso ponto de corte. Sétimo, o valor AIP para o subgrupo Rentrop 1 apresentou uma inconsistência causada por valores discrepantes que deve ser interpretada com cautela. Oitavo, embora a validação interna por *bootstrap* tenha confirmado a estabilidade do modelo (AUC corrigida para otimismo = 0,967), é necessária a validação externa em coortes independentes. Em nono lugar, a regressão logística ordinal usando o desfecho completo de Rentrop de 4 graus confirmou os achados, mas foi limitada pelo tamanho da amostra nas categorias extremas de Rentrop. Em décimo lugar, o alto FIV para IPN (19,38) no modelo final indica colinearidade com a Escore HALP; análises de sensibilidade com um modelo de duas variáveis confirmaram a robustez.

## Conclusão

Este estudo demonstra, pela primeira vez, que o escore HALP está associado de forma independente à CCC em pacientes com SCC e oclusão coronariana total. A excelente capacidade discriminatória (AUC = 0,958), confirmada pela validação interna por *bootstrap* (AUC corrigida para otimismo = 0,967), aliada à simplicidade e custo-efetividade, apoia o escore HALP como um candidato promissor para estratificação de risco, aguardando validação prospectiva. Nossos achados ressaltam a primazia da inflamação, do estado nutricional e da função imunológica — em vez dos fatores de risco tradicionais baseados em lipídios — na regulação da capacidade arteriogênica. Estudos prospectivos futuros são necessários para validar esses achados e explorar o potencial terapêutico da modulação dos componentes do escore HALP para aprimorar a colateralização coronariana.

### Mensagens principais

- O escore HALP — que integra hemoglobina, albumina, linfócitos e plaquetas — está associado de forma independente à CCC em pacientes com OTC, com escores mais altos prevendo melhor colateralização (AUC=0,958; AUC corrigida para otimismo=0,967).
- O estado inflamatório-nutricional sistêmico, em vez dos fatores de risco tradicionais baseados em lipídios, parece ser o principal determinante da capacidade arteriogênica nessa população.
- Um valor de corte do escore HALP >49,08 identifica pacientes com alta probabilidade de apresentarem circulação colateral bem desenvolvida, com alta sensibilidade (91,3%) e especificidade (90,8%), sugerindo potencial utilidade para estratificação de risco, aguardando validação prospectiva.
