# Supplementary material for: O Escore HALP como um Novo Marcador Associado à Circulação Colateral Coronariana em Pacientes com Oclusão Total Crônica
Source: Arq Bras Cardiol. 2026 Aug 3;123(7):e20260206. [Article in Portuguese] doi: 10.36660/abc.20260206 (PMC13668459; doi:10.36660/abc.20260206)
Supplement: Translation

# Translation: HALP Score as a Novel Marker Associated with Coronary Collateral Circulation in Patients with Chronic Total Occlusion

**DOI:** 10.36660/abc.20260206i

**Authors:** Hasan Akkaya, Muhammet Öztürk

**Affiliations:** 1 Türkiye Department of Cardiology, Niğde Training and Research Hospital, Niğde – Türkiye

**Keywords:** Collateral Circulation, Coronary Occlusion, Prognosis, Inflammation

## Abstract

**Background:**

Coronary collateral circulation (CCC) is a key determinant of myocardial viability and prognosis in patients with chronic total occlusion (CTO). The hemoglobin, albumin, lymphocyte, and platelet (HALP) score reflects nutritional status, immune competence, and systemic inflammation.

**Objectives:**

To investigate the association between HALP score and CCC in patients with CTO.

**Methods:**

This retrospective, single-center study included 269 patients with chronic coronary syndrome and angiographically confirmed CTO. Patients were classified according to Rentrop grades and dichotomized into poor CCC (Rentrop 0–1; n=119) and good CCC (Rentrop 2–3; n=150). HALP score, inflammatory markers, atherogenic indices, and angiographic scores were compared between groups. Multivariate logistic regression and receiver operating characteristic (ROC) analyses were performed. A p-value <0.05 was considered statistically significant.

**Results:**

HALP score increased significantly across Rentrop grades (p<0.001) and was higher in patients with good CCC than in those with poor CCC (75.52±13.91 vs. 35.10±11.29; p<0.001). In multivariable analysis, HALP score (OR:0.88, 95% CI: 0.83–0.93; p<0.001), Gensini score (OR:1.14; p=0.002), and Prognostic Nutritional Index (OR:0.73; p=0.003) were independent predictors of good CCC. ROC analysis showed high discriminative ability (AUC=0.958; 95% CI: 0.93–0.98), with a cut-off value of >49.08 (91.3% sensitivity, 90.8% specificity). Atherogenic lipid indices were not significantly associated with CCC.

**Conclusions:**

HALP score is an independent and readily available marker associated with CCC in CTO patients and may serve as a practical tool for risk stratification, highlighting the importance of inflammatory, nutritional, and immune pathways in collateral development.

## Introduction

Coronary artery disease remains the leading global cause of cardiovascular mortality, with chronic total occlusion (CTO) representing a particularly challenging subset occurring in 15–30% of patients undergoing diagnostic angiography.^1,2^ CTO revascularization remains technically demanding, and many patients receive optimal medical therapy alone; therefore, coronary collateral circulation (CCC) adequacy becomes a critical determinant of myocardial viability and long-term prognosis.^3,4^

CCC encompasses pre-existing or newly formed vascular conduits bypassing occluded segments, with marked interindividual variability ranging from virtually absent (Rentrop 0) to complete compensatory flow (Rentrop 3).^5^ Well-developed collaterals confer smaller infarct sizes, better left ventricular function preservation, and improved survival,^6-8^ whereas inadequate CCC associates with larger ischemic areas and increased mortality.^9^

Coronary collateral formation occurs primarily through arteriogenesis—remodeling and enlargement of pre-existing arteriolar connections driven by hemodynamic shear stress from pressure gradients across stenotic segments.^10^ However, arteriogenesis is profoundly influenced by systemic inflammatory status, immune function, nutritional state, and hematological parameters.^1 1^ Chronic low-grade inflammation impairs endothelial function and inhibits collateral formation,^1 2^ whereas balanced immune responses promote endothelial repair.^1 3^ Additionally, malnutrition and hypoalbuminemia compromise vascular integrity, while anemia limits oxygen delivery, attenuating hypoxia-inducible factor pathways.^14,15^

Inflammatory indices and nutritional markers have been associated with coronary outcomes,^16-20^ and prior studies linked Neutrophil-to-Lymphocyte Ratio **(**NLR) and Platelet-to-Lymphocyte Ratio **(**PLR) to CCC.^2 1,2 2^ However, these single-parameter indices capture only partial aspects of the inflammatory-nutritional interplay governing arteriogenesis.

The HALP score—calculated as (Hemoglobin × Albumin × Lymphocyte count) / Platelet count—integrates four key components reflecting oxygen-carrying capacity, nutritional status, immune competence, and hemostatic-inflammatory activity.^23,24^ Recently, the HALP score demonstrated cardiovascular relevance, with lower scores independently associated with higher acute myocardial infarction mortality^25^ and adverse events post-intervention.^26^ However, no prior study investigated the HALP score’s relationship with coronary collateralization.

Traditional lipid-based cardiovascular risk relies on parameters including low-density lipoprotein cholesterol, high-density lipoprotein cholesterol, and triglycerides. Emerging evidence suggests composite atherogenic indices—including Atherogenic Index of Plasma, Castelli Risk Indices, Atherogenic Coefficient, and Triglyceride-Glucose Index—may better reflect metabolic dysregulation.^27,28^ However, their association with coronary collateral development remains poorly characterized, leaving unresolved whether lipid-driven atherogenesis directly influences arteriogenic capacity or whether collateralization is governed primarily by hemodynamic and inflammatory factors independent of lipid metabolism.

Given CCC’s critical role in CTO patients and the paucity of integrative biomarkers, we designed this study to comprehensively evaluate associations between HALP score, inflammatory parameters, atherogenic indices, and coronary collateralization. To our knowledge, this represents the first study investigating the HALP score’s association with CCC.

## Methods

### Study design and population

This retrospective, observational, single-center study was conducted at Blinded for Review. The study protocol received approval from the Blinded for Review Institutional Ethics Committee (and adhered to Declaration of Helsinki principles. Given the retrospective nature of utilizing anonymized data, informed consent requirements were waived.

Patients aged ≥18 years diagnosed with chronic coronary syndrome (CCS) per current guidelines^29^ who underwent coronary angiography between August 2021 and January 2026 were screened. Inclusion criteria comprised: typical angina, electrocardiographic ischemia evidence, positive myocardial perfusion scintigraphy, or critical stenosis on coronary computed tomographic angiography; plus angiographically documented total occlusion (100% stenosis, TIMI 0 flow) in ≥1 major epicardial coronary artery.

Exclusion criteria encompassed: acute coronary syndrome within the preceding 6 months; prior coronary bypass grafting; moderate-to-severe valvular disease; eGFR <30 ml/min/1.73m^2^; active hepatic disease; known active malignancy; NYHA class III–IV heart failure; moderate-to-severe chronic obstructive pulmonary disease; acute/chronic infectious or rheumatologic disease; or incomplete data.

### Data collection and laboratory assessment

Baseline demographic, clinical, laboratory, and angiographic data were retrospectively extracted from electronic records. Cardiovascular risk factors and medications were documented at the time of angiography.

Venous blood samples were obtained after overnight fasting (≥8 hours) on angiography mornings. Laboratory parameters included: complete blood count (hemoglobin, RDW, WBC, neutrophil, lymphocyte, monocyte, platelet); biochemical parameters (fasting glucose, creatinine, AST, ALT, hsCRP, HbA1c, total cholesterol, HDL-C, LDL-C, triglycerides, albumin). eGFR was calculated using the CKD-EPI equation.

### Calculation of composite indices

- **HALP score** = (Hemoglobin [g/L] × Albumin [g/dL] × Lymphocyte count [×10^9^/L]) / Platelet count (×10^9^/L).
- **CALLY Index** = Albumin × Lymphocyte count / C-reactive protein
- **Systemic Inflammation Response Index (SIRI)** = Neutrophil count × Monocyte count / Lymphocyte count
- **Systemic Immuno-Inflammation Index (SII)** = Platelet count × Neutrophil count / Lymphocyte count
- **Prognostic Nutritional Index (PNI)** = 10 × Albumin + 0.005 × Lymphocyte count
- **Platelet-to-Lymphocyte Ratio (PLR)** = Platelet count / Lymphocyte count
- **Neutrophil-to-Lymphocyte Ratio (NLR)** = Neutrophil count / Lymphocyte count
- **Monocyte-to-Lymphocyte Ratio (MLR)** = Monocyte count / Lymphocyte count
- **Pan-Immune Inflammation Value (PIV)** = Neutrophil × Platelet × Monocyte / Lymphocyte
- **Atherogenic Index of Plasma (AIP)** = Log₁₀ (Triglycerides / HDL cholesterol)
- **Castelli Risk Index I (CRI-I)** = Total cholesterol / HDL cholesterol
- **Castelli Risk Index II (CRI-II)** = LDL cholesterol / HDL cholesterol
- **Atherogenic Coefficient (AC)** = (Total cholesterol − HDL cholesterol) / HDL cholesterol
- **Triglyceride-Glucose Index (TyG)** = Ln (Triglycerides × Fasting glucose / 2)

### Coronary angiography and collateral assessment

Coronary angiography was performed via femoral or radial approach. Two experienced interventional cardiologists, blinded to clinical-laboratory data, reviewed angiographic images; disagreements were adjudicated by a third senior cardiologist. SYNTAX Score and Gensini Score were calculated using standard methods. Coronary collateral circulation was evaluated using Rentrop grading (0–3).^5^ Patients were dichotomized into poor CCC (Rentrop 0–1) and good CCC (Rentrop 2–3) for secondary analyses.

### Statistical analysis

Statistical analyses were performed using IBM SPSS Statistics version 22.0 and MedCalc version 22.018. Two-tailed p<0.05 was considered statistically significant. Continuous variables were tested for normality using the Shapiro-Wilk test. Normally distributed variables are presented as mean±SD; categorical variables as frequencies and percentages. Categorical variables were compared using χ^2^ or Fisher’s exact test. Continuous variables were compared using an independent samples t-test or one-way ANOVA with Bonferroni post-hoc correction. The Pearson correlation coefficient assessed linear associations between continuous variables. Univariate binary logistic regression identified variables associated with good CCC. Variables with p<0.10 in univariate analysis, plus clinically relevant covariates (sex, atrial fibrillation, left ventricular ejection fraction (LVEF)), were entered into multivariable binary logistic regression using the enter method. Sex, atrial fibrillation, and LVEF were included as covariates given their significant associations with Rentrop staging in baseline analysis (p=0.004, p=0.001, and p=0.005, respectively), reflecting known biological relationships between these variables and arteriogenic capacity. Multicollinearity was assessed using the variance inflation factor (VIF); VIF >5 indicated significant multicollinearity. VIF values for the final three-variable model were: HALP score 7.13, Gensini score 7.90, and PNI 19.38. Although PNI showed a VIF >5, it was retained given its established biological relevance and independent significance; sensitivity analyses with a two-variable model (HALP + Gensini) confirmed robustness of findings. Model goodness-of-fit was evaluated using the Hosmer-Lemeshow test (p>0.05 indicating good fit). Results are presented as odds ratios with 95% confidence intervals. ROC curve analysis assessed the HALP score’s discriminatory ability for predicting good CCC. Internal validation was performed using bootstrap resampling (1,000 iterations). The optimism-corrected AUC was 0.967 (95%CI 0.958–0.969), confirming model stability and absence of meaningful overfitting. Optimal cut-off was determined using Youden’s index.

## Results

### Baseline characteristics

A total of 347 patients with CTO undergoing coronary angiography for CCS were screened. Seventy-eight patients were excluded due to acute coronary syndrome within 6 months (n=18), prior coronary artery bypass grafting (CABG) (n=12), estimated glomerular filtration rate (eGFR) <30 mL/min/1.73 m^2^ (n=15), active malignancy (n=8), or incomplete data (n=25). The remaining 269 patients with CCS and ≥1 totally occluded coronary artery were included (see Figure 1 for patient flow diagram). Patients were stratified by Rentrop grading: Rentrop 0 (n=30, 11.2%), Rentrop 1 (n=89, 33.1%), Rentrop 2 (n=105, 39.0%), and Rentrop 3 (n=45, 16.7%). For further analysis, patients were dichotomized into poor CCC (Rentrop 0–1; n=119, 44.2%) and good CCC (Rentrop 2–3; n=150, 55.8%).

**Figure 1:** – Patient flow diagram. CCC: Coronary collateral circulation.

Baseline demographic and clinical characteristics across Rentrop stages are presented in Table 1. Significant associations were observed between Rentrop stages and sex (p=0.004) and atrial fibrillation prevalence (p=0.001). Detailed group comparisons are presented in Table 1. No significant differences were observed regarding diabetes mellitus, hypertension, dyslipidemia, current smoking, or medication use (p>0.05 for all).

**Table 1:** – Baseline demographic and clinical characteristics according to Rentrop Stages

Characteristic	Rentrop 0 (n=30)	Rentrop 1 (n=89)	Rentrop 2 (n=105)	Rentrop 3 (n=45)	p
**Age (years)**	72.83±9.50*	68.17±11.54	64.75±10.29	62.44±10.26	<0.001
**Male sex, n (%)**	19 (63.3)	59 (66.3)	86 (81.9)	40 (88.9)	0.004
**BMI (kg/m^2^)**	28.22±4.63	27.64±3.59	26.91±3.53	27.61±3.35	0.272
**Risk factors, n (%)**					
Diabetes mellitus	12 (40.0)	34 (38.2)	36 (34.3)	16 (35.6)	0.917
Hypertension	20 (66.7)	52 (58.4)	61 (58.1)	24 (53.3)	0.724
Dyslipidemia	14 (46.7)	36 (40.4)	52 (49.5)	18 (40.0)	0.554
Current smoking	12 (40.0)	38 (42.7)	41 (39.0)	27 (60.0)	0.113
Atrial fibrillation	5 (16.7)	9 (10.1)	4 (3.8)	0 (0.0)	0.001
**Medications, n (%)**					
Beta-blocker	20 (66.7)	53 (59.6)	57 (54.3)	26 (57.8)	0.658
ACE-I/ARB	14 (46.7)	35 (39.3)	47 (44.8)	20 (44.4)	0.844
Statin	12 (40.0)	24 (27.0)	34 (32.4)	14 (31.1)	0.595
Calcium channel blocker	9 (30.0)	18 (20.2)	19 (18.1)	8 (17.8)	0.523

Data are presented as mean±standard deviation or n (%). P values: one-way ANOVA or χ^2^ test. *p<0.05 vs Rentrop 2 and Rentrop 3 (Bonferroni correction). BMI: body mass index; ACE-I: angiotensin-converting enzyme inhibitor; ARB: angiotensin receptor blocker.

### Laboratory and angiographic parameters

Comparative analysis across Rentrop stages revealed significant differences in multiple clinical, hematological, inflammatory, and angiographic parameters (Table 2). Key clinical differences are summarized in Table 2. Notably, age, LVEF, and eGFR showed significant differences across Rentrop stages (p<0.001, p=0.005, and p=0.022, respectively), while biochemical parameters showed no significant differences (p>0.05 for all).

**Table 2:** – Laboratory, Hematological, Inflammatory Parameters, and Angiographic Scores According to Rentrop Stages

Characteristic	Rentrop 0 (n=30)	Rentrop 1 (n=89)	Rentrop 2 (n=105)	Rentrop 3 (n=45)	p
**Clinical/Biochemical**					
LVEF (%)	45.80±10.15*	49.19±8.27	50.58±6.48	51.82±6.38	0.005
eGFR (ml/min/1.73m^2^)	71.33±16.99†	77.55±14.63	82.69±13.67	87.12±14.95	0.022
Creatinine (mg/dL)	1.21±0.91	1.05±0.50	1.03±0.51	0.97±0.30	0.338
AST (U/L)	27.57±11.27	24.27±9.67	23.77±9.28	23.00±7.58	0.391
ALT (U/L)	19.37±9.54	19.94±8.65	19.95±9.14	18.78±8.81	0.917
Albumin (g/dL)	38.82±3.46	40.51±3.42	42.57±2.76	43.36±2.82‡	<0.001
hsCRP (mg/L)	5.95±4.59	5.46±1.49	3.97±4.58	4.76±7.56	0.131
**Hematological**					
Hemoglobin (g/L)	12.35±2.36	13.68±1.89	14.56±1.84	14.92±1.36‡	<0.001
WBC (×10^9^/L)	9.24±2.85	9.08±2.98	9.02±2.52	9.51±2.32	0.770
Neutrophil (×10^9^/L)	6.84±2.49	6.38±2.60	5.72±2.23	5.05±1.37*	0.002
Lymphocyte (×10^9^/L)	1.61±0.59	1.94±0.73	2.43±0.72	3.49±1.29‡	<0.001
Monocyte (×10^9^/L)	0.61±0.25	0.60±0.24	0.66±0.23	0.73±0.22	0.015
Platelet (×10^9^/L)	300.40±58.65*	272.06±47.63	243.47±51.33	225.47±53.74	<0.001
**Inflammatory/Nutritional indices**					
HALP score	24.10±11.30	38.81±10.44	64.73±15.91	100.70±16.37‡	<0.001
CALLY index	26.07±12.87	26.70±11.55	53.54±13.45	99.31±18.31‡	<0.001
SII	1837.27±329.81	1042.91±261.02	646.64±126.33	361.26±59.66	<0.001
PNI	37.73±7.32	40.04±6.51	42.65±2.76	43.29±2.85	<0.001
PLR	246.02±15.62	149.49±14.98	109.61±12.11	72.00±10.64‡	<0.001
NLR	6.03±2.71‡	3.66±1.55	2.82±0.35	1.72±0.87	<0.001
MLR	0.48±0.21‡	0.34±0.14	0.32±0.12	0.23±0.09	0.001
SIRI	3.65±0.37	2.54±0.24	2.18±1.41	1.19±0.63	0.077
PIV	1089.06±157.96	773.98±106.71	512.71±89.36	265.74±62.67	0.047
**Atherogenic indices**					
AIP	0.15±0.11	0.21±0.18	0.26±0.14	0.24±0.11	0.577
CRI-I	4.46±1.20	4.69±1.03	4.83±1.17	4.98±1.41	0.223
CRI-II	2.81±0.47	2.98±0.94	2.97±0.96	3.04±1.20	0.805
TyG index	9.18±0.71	9.24±0.59	9.30±0.58	9.21±0.53	0.700
**Angiographic scores**					
SYNTAX score	26.58±7.24‡	22.71±6.59	18.12±5.61	16.84±6.78	<0.001
Gensini score	59.60±18.87	53.09±16.07*	36.87±12.11	31.49±13.77	<0.001

OData are presented as mean±standard deviation. P values: one-way ANOVA with Bonferroni post-hoc correction. *p<0.05 vs Rentrop 3; †p<0.05 vs Rentrop 3; ‡p<0.05 vs all other groups. LVEF: left ventricular ejection fraction; eGFR: estimated glomerular filtration rate; AST: aspartate aminotransferase; ALT: alanine aminotransferase; hsCRP: high-sensitivity C-reactive protein; WBC: white blood cell; SII: systemic immuno-inflammation index; PNI: prognostic nutritional index; PLR: platelet-to-lymphocyte ratio; NLR: neutrophil-to-lymphocyte ratio; MLR: monocyte-to-lymphocyte ratio; SIRI: systemic inflammation response index; PIV: pan-immune inflammation value; AIP: atherogenic index of plasma; CRI: Castelli risk index; TyG: triglyceride-glucose index.

Hematological parameters showed marked stepwise differences across Rentrop stages (Table 2). Albumin, hemoglobin, and lymphocyte counts progressively increased with better collateralization, while neutrophil and platelet counts decreased (all p<0.001).

HALP score demonstrated a striking stepwise gradient across Rentrop stages (p<0.001), with Rentrop 3 showing significantly higher values versus all other groups. CALLY index and PNI showed similar stepwise increases (Table 2).

Inflammatory indices showed inverse relationships with collateralization. SII, PLR, NLR, MLR, and PIV were all significantly higher in poor CCC patients (Table 2). SIRI showed a trend toward reduction with better collateralization (p=0.077).

Angiographic complexity was greater in poor CCC patients, with higher SYNTAX and Gensini scores (Table 2; p<0.001 for both).

Atherogenic lipid indices, including AIP, CRI-I, CRI-II, AC, and TyG index, showed no significant differences across Rentrop stages (p>0.05 for all), suggesting traditional lipid-based cardiovascular risk factors do not independently influence coronary collateral development.

### Poor vs. good CCC comparison

Consistent with previous literature, coronary collateral circulation was categorized into poor CCC (Rentrop 0–1; n=119) and good CCC (Rentrop 2–3; n=150). Detailed comparisons are presented in Table 3. Good CCC patients had significantly higher HALP, CALLY, and PNI scores, while inflammatory markers (SII, PLR, NLR, MLR, PIV, and hsCRP/Albumin ratio) were significantly elevated in poor CCC patients (all p<0.05).

**Table 3:** – Comparison of Parameters between Poor and Good Coronary Collateral Circulation Groups

Parameter	Poor CCC (n=119)	Good CCC (n=150)	p
**Angiographic scores**			
SYNTAX score	23.68±6.73	17.74±6.08	<0.001
Gensini score	54.73±16.97	35.25±13.14	<0.001
**Inflammatory and nutritional indices**			
HALP score	35.10±11.29	75.52±13.91	<0.001
CALLY index	26.67±4.57	68.30±12.63	<0.001
SIRI	2.82±1.44	1.88±0.39	0.065
SII	1243.17±35.88	561.03±25.99	<0.001
PNI	39.46±5.81	42.84±2.80	<0.001
PLR	173.82±10.04	98.33±8.57	<0.001
NLR	4.26±1.83	2.49±0.66	<0.001
MLR	0.37±0.18	0.29±0.11	0.014
hsCRP/Albumin ratio	0.14±0.06	0.10±0.02	0.007
PIV	853.41±99.14	438.62±63.91	0.016
**Atherogenic indices**			
AIP	2.84±0.25	0.26±0.05	0.331
CRI-I	4.63±1.07	4.88±1.25	0.083
CRI-II	2.94±0.94	2.99±1.04	0.657
AC	3.61±1.06	3.86±1.25	0.076
TyG index	9.22±0.62	9.27±0.57	0.492

Data are presented as mean±standard deviation. P values: independent samples t-test. CCC: coronary collateral circulation; SIRI: systemic inflammation response index; SII: systemic immuno-inflammation index; PNI: prognostic nutritional index; PLR: platelet-to-lymphocyte ratio; NLR: neutrophil-to-lymphocyte ratio; MLR: monocyte-to-lymphocyte ratio; hsCRP: high-sensitivity C-reactive protein; PIV: pan-immune inflammation value; AIP: atherogenic index of plasma; CRI: Castelli risk index; AC: atherogenic coefficient; TyG: triglyceride-glucose index.

Angiographic complexity was greater in poor CCC patients (Table 3). Atherogenic indices did not differ significantly between groups (p>0.05 for all).

### Correlation analysis

Pearson correlation analysis (Table 4) revealed significant associations between the HALP score and multiple parameters. HALP score demonstrated strong negative correlations with PLR (r=−0.670, p<0.001), moderate negative correlations with Gensini score (r=−0.517), SII (r=−0.451), SYNTAX score (r=−0.439), and NLR (r=−0.418), and weak negative correlations with MLR, PIV, and SIRI (all p<0.001). Positive correlations were observed with CALLY index (r=+0.413) and PNI (r=+0.292). Detailed values are presented in Table 4.

**Table 4:** –Correlation between HALP Score and Study Parameters

Parameter	r	p
**Angiographic scores**		
SYNTAX score	–0.439	<0.001
Gensini score	–0.517	<0.001
**Inflammatory and nutritional indices**		
CALLY index	+0.413	<0.001
SIRI	–0.209	0.001
SII	–0.451	<0.001
PNI	+0.292	<0.001
PLR	–0.670	<0.001
NLR	–0.418	<0.001
MLR	–0.294	<0.001
PIV	–0.232	<0.001
hsCRP/Albumin ratio	–0.105	0.087
**Atherogenic indices**		
AIP	–0.075	0.218
CRI-I	+0.123	0.043
CRI-II	+0.088	0.151
AC	+0.125	0.040
TyG index	–0.020	0.745

Pearson correlation analysis. SIRI: systemic inflammation response index; SII: systemic immuno-inflammation index; PNI: prognostic nutritional index; PLR: platelet-to-lymphocyte ratio; NLR: neutrophil-to-lymphocyte ratio; MLR: monocyte-to-lymphocyte ratio; PIV: pan-immune inflammation value; hsCRP: high-sensitivity C-reactive protein; AIP: atherogenic index of plasma; CRI: Castelli risk index; AC: atherogenic coefficient; TyG: triglyceride-glucose index.

### Logistic regression analysis

Univariate logistic regression identified multiple parameters significantly associated with good CCC development (Table 5). After adjusting for potential confounders in the multivariable model, only three parameters remained independent associates of good CCC: HALP score (OR 0.88, 95%CI 0.83–0.93, p<0.001), Gensini score (OR 1.14, 95%CI 1.05–1.24, p=0.002), and PNI (OR 0.73, 95%CI 0.60–0.90, p=0.003).

**Table 5:** – Univariate and multivariable logistic regression analysis for predictors of good coronary collateral circulation

Variable	Univariate OR (95% CI)	p	Multivariate OR (95% CI)	p
HALP score	0.86 (0.83–0.89)	<0.001	0.88 (0.83–0.93)	<0.001
Gensini score	1.08 (1.06–1.10)	<0.001	1.13 (1.05–1.22)	0.002
PNI	0.76 (0.69–0.83)	<0.001	0.73 (0.60–0.90)	0.003
Age (years)	1.04 (1.02–1.07)	<0.001	1.02 (0.97–1.07)	0.388
SYNTAX score	1.15 (1.10–1.20)	<0.001	0.85 (0.69–1.04)	0.128
CALLY index	0.98 (0.97–0.99)	<0.001	0.99 (0.98–1.01)	0.515
SII	1.00 (0.99–1.03)	<0.001	1.00 (0.99–1.01)	0.084
PLR	1.03 (1.02–1.04)	<0.001	0.99 (0.97–1.02)	0.676
NLR	1.42 (1.22–1.66)	<0.001	0.57 (0.21–1.53)	0.261
hsCRP/Albumin ratio	19.67 (2.05–188.98)	0.009	27.06 (0.17–44.61)	0.201
SIRI	1.13 (0.97–1.31)	0.102	—	—
AIP	1.01 (0.96–1.07)	0.602	—	—

Dependent variable: Poor coronary collateral circulation (Rentrop 0-1=1, good CCC=0). Bold values indicate independent predictors in multivariable analysis. OR<1 for HALP score indicates that higher HALP scores are associated with reduced odds of poor CCC (protective effect). VIF values for final model: HALP=7.13, Gensini=7.90, PNI=19.38. Bootstrap internal validation (1,000 iterations): optimism-corrected AUC=0.958 (95%CI: 0.929-0.980). OR: odds ratio; CI: confidence interval; PNI: prognostic nutritional index; SII: systemic immuno-inflammation index; PLR: platelet-to-lymphocyte ratio; NLR: neutrophil-to-lymphocyte ratio; SIRI: systemic inflammation response index; hsCRP: high-sensitivity C-reactive protein; AIP: atherogenic index of plasma.

The dependent variable was coded as good CCC = 1 (Rentrop 2–3) and poor CCC = 0 (Rentrop 0–1). The OR of 0.88 for the HALP score indicates that for each one-unit increase in HALP score, the odds of being classified as good CCC increase (i.e., the odds of poor CCC decrease by 12%). This is directionally consistent with the biological hypothesis: higher HALP scores are associated with better collateralization. The apparent inverse OR reflects the scale of the HALP variable relative to the binary outcome coding.

An additional ordinal logistic regression using the full 4-grade Rentrop outcome confirmed the independent association of HALP score (OR 0.91, 95%CI 0.87–0.95, p<0.001), Gensini score (OR 1.09, 95%CI 1.02–1.17, p=0.012), and PNI (OR 0.81, 95%CI 0.68–0.96, p=0.016) with Rentrop grade, supporting the robustness of findings across the full ordinal spectrum.

### ROC analysis

ROC curve analysis demonstrated HALP score possessed excellent discriminatory power for predicting good CCC (Figure 2). AUC was 0.958 (95%CI 0.93–0.98, p<0.001). Internal validation using bootstrap resampling (1,000 iterations) yielded an optimism-corrected AUC of 0.967 (95%CI 0.958–0.969), confirming model stability and absence of meaningful overfitting. Optimal cut-off >49.08 yielded sensitivity 91.3% (95%CI 85.6–95.3%) and specificity 90.8% (95%CI 84.1–95.3%).

**Figure 2:** – Receiver operating characteristic curve for HALP Score in Predicting Good Coronary Collateral Circulation.

## Discussion

This study investigated relationships between HALP score, inflammatory parameters, atherogenic indices, and coronary collateral circulation in CTO patients. Our principal findings demonstrate: (1) HALP score exhibited robust stepwise increase across Rentrop stages; (2) inflammatory indices were significantly elevated in poor CCC patients; (3) atherogenic lipid indices did not differ across Rentrop stages; (4) in multivariable analysis, HALP score, Gensini score, and PNI emerged as independent associates of good CCC; and (5) HALP score cut-off >49.08 demonstrated excellent discriminatory ability (AUC=0.958), confirmed by bootstrap internal validation (optimism-corrected AUC=0.967). A visual summary is presented in the Central Illustration.

HALP score integrates hemoglobin (oxygen-carrying capacity), albumin (nutritional status and anti-inflammatory properties), lymphocytes (adaptive immune competence), and platelets (hemostatic-inflammatory activity). Higher hemoglobin supports angiogenic activity via HIF-1α/VEGF pathways,^30^ while adequate albumin preserves endothelial function and nitric oxide bioavailability.^15,31^ Lymphocytes orchestrate vascular repair through cytokine-mediated endothelial progenitor cell mobilization,^1 3^ and lower platelet counts in well-collateralized patients may reflect reduced chronic inflammatory activation.^32^

Limited studies have explored the HALP score in cardiovascular contexts. Xu et al.25 reported that lower HALP scores were independently associated with higher acute myocardial infarction mortality, while Liu et al.26 demonstrated that the HALP score predicted the no-reflow phenomenon and long-term prognosis in patients with ST-segment elevation myocardial infarction after primary percutaneous coronary intervention. Our findings extend these observations to coronary collateral development. The multivariable-adjusted OR of 0.88 (95%CI 0.83–0.93) indicates each unit increase in HALP score confers 12% reduction in poor CCC likelihood.

Multiple inflammatory indices were inversely associated with collateral development. SII, NLR, PLR, and PIV were all significantly elevated in poor CCC patients, consistent with evidence that heightened systemic inflammation impairs endothelial function and inhibits arteriogenesis.^1 2^ Our results corroborate prior findings by Açar et al.^2 1^ and Kurtul et al.,^2 2^ extending these observations by demonstrating HALP score may serve as a more integrative marker capturing both inflammatory burden and nutritional-immune reserve.

PNI emerged as an independent good CCC associate in our multivariable model (OR 0.73, 95%CI 0.60–0.90, p=0.003), consistent with recent data from Keskin et al.^20^ The absence of significant associations between atherogenic lipid indices and CCC suggests arteriogenesis is primarily governed by hemodynamic-inflammatory rather than lipid-based mechanisms,^10^ supported by animal studies showing hypercholesterolemia does not necessarily impair collateral growth.^33^

Gensini score emerged as an independent good CCC associate (OR 1.14, 95%CI 1.05–1.24, p=0.002), consistent with the concept that chronic progressive stenosis creates sustained hemodynamic gradients stimulating arteriogenesis.^8^ Gensini score, accounting for lesion severity and extent, may better capture atherosclerosis chronicity compared to the SYNTAX score.

HALP score, as a simple and readily calculable biomarker, may represent a useful adjunct for risk stratification in CTO patients. Patients with lower HALP scores may represent a higher-risk subgroup warranting closer follow-up; however, given the observational and retrospective nature of this study, these findings should be considered hypothesis-generating and require prospective validation before informing clinical decision-making.

Our findings suggest interventions aimed at improving individual HALP score components may promote collateralization. Nutritional supplementation (to raise albumin levels), anemia correction (to optimize hemoglobin), immunomodulatory therapies (to enhance lymphocyte function), and antiplatelet strategies balanced to avoid excessive platelet suppression may collectively enhance arteriogenic capacity. Preliminary evidence from randomized trials suggests granulocyte-macrophage colony-stimulating factor administration can augment collateral development by mobilizing monocytes and endothelial progenitor cells,^34^ though larger confirmatory studies are needed.

Our study underscores the critical interplay between inflammation, nutritional status, and immune function in governing cardiovascular remodeling. This paradigm shift—from viewing atherosclerosis solely as a lipid-driven disease to recognizing it as an inflammatory-immune disorder—has profound therapeutic development implications. Emerging anti-inflammatory agents such as canakinumab (anti-IL-1β) and low-dose methotrexate showed promise in reducing cardiovascular events,^35^ and our findings suggest they may exert salutary effects by preserving collateral function.

### Limitations

Several limitations warrant acknowledgment. First, the retrospective, single-center design may limit generalizability and introduce selection bias. Second, coronary collateral assessment was based solely on angiographic Rentrop grading. Third, we did not serially measure the HALP score over time, precluding temporal dynamics and causality analysis. Fourth, residual confounding cannot be entirely excluded despite multivariable adjustment. Fifth, the study population was limited to CTO patients. Sixth, albumin units (g/dL) used in our HALP formula differ from some published formulas using g/L; readers should apply the same unit convention when using our reported cut-off. Seventh, the AIP value for the Rentrop 1 subgroup showed an outlier-driven inconsistency that should be interpreted with caution. Eighth, although bootstrap internal validation confirmed model stability (optimism-corrected AUC=0.967), external validation in independent cohorts is needed. Ninth, ordinal logistic regression using the full 4-grade Rentrop outcome confirmed findings but was limited by sample size in extreme Rentrop categories. Tenth, the high VIF for PNI (19.38) in the final model indicates collinearity with HALP score; sensitivity analyses with a two-variable model confirmed robustness.

## Conclusion

This study demonstrates for the first time that the HALP score is independently associated with coronary collateral circulation in CCS patients with total coronary occlusion. The excellent discriminatory ability (AUC=0.958), confirmed by bootstrap internal validation (optimism-corrected AUC=0.967), coupled with simplicity and cost-effectiveness, supports the HALP score as a promising candidate for risk stratification, pending prospective validation. Our findings underscore the primacy of inflammation, nutritional status, and immune function—rather than traditional lipid-based risk factors—in governing arteriogenic capacity. Future prospective studies are warranted to validate these findings and explore the therapeutic potential of modulating HALP score components to enhance coronary collateralization.

### Key messages

- HALP score—integrating hemoglobin, albumin, lymphocyte, and platelet—is independently associated with coronary collateral circulation in chronic total occlusion patients, with higher scores predicting better collateralization (AUC=0.958; optimism-corrected AUC=0.967).
- Systemic inflammatory-nutritional status, rather than traditional lipid-based risk factors, appears to be the dominant determinant of arteriogenic capacity in this population.
- A HALP score cut-off >49.08 identifies patients likely to have well-developed collaterals with high sensitivity (91.3%) and specificity (90.8%), suggesting potential utility for risk stratification pending prospective validation.
